# A framework for conceptualising early intervention for eating disorders

**DOI:** 10.1002/erv.2959

**Published:** 2022-11-25

**Authors:** Karina L. Allen, Victoria A. Mountford, Rosiel Elwyn, Michaela Flynn, Anthea Fursland, Nicole Obeid, Georgina Partida, Katie Richards, Ulrike Schmidt, Lucy Serpell, Scout Silverstein, Tracey Wade

**Affiliations:** ^1^ Eating Disorders Outpatients Service Maudsley Hospital South London and Maudsley NHS Foundation Trust London UK; ^2^ Department of Psychological Medicine Institute of Psychiatry, Psychology and Neuroscience King's College London London UK; ^3^ LightHouse Arabia Dubai United Arab Emirates; ^4^ Thompson Institute University of the Sunshine Coast Gubbi Gubbi Country Queensland Australia; ^5^ The Swan Centre Perth Western Australia Australia; ^6^ Children's Hospital of Eastern Ontario Research Institute Ottawa Ontario Canada; ^7^ Department of Clinical, Educational and Health Psychology University College London London UK; ^8^ Eating Disorder Service North East London NHS Foundation Trust Essex UK; ^9^ Equip Health Carlsbad California USA; ^10^ Flinders Institute for Mental Health and Wellbeing Flinders University Adelaide South Australia Australia

**Keywords:** anorexia nervosa, bulimia nervosa, early intervention, eating disorders, mental health

## Abstract

**Objective:**

This paper outlines the evidence base for early intervention for eating disorders; provides a global overview of how early intervention for eating disorders is provided in different regions and settings; and proposes policy, service, clinician and research recommendations to progress early intervention for eating disorders.

**Method and Results:**

Currently, access to eating disorder treatment often takes many years or does not occur at all. This is despite neurobiological, clinical and socioeconomic evidence showing that early intervention may improve outcomes and facilitate full sustained recovery from an eating disorder. There is also considerable variation worldwide in how eating disorder care is provided, with marked inequalities in treatment provision. Despite these barriers, there are existing evidence‐based approaches to early intervention for eating disorders and progress is being made in scaling these.

**Conclusions:**

We propose action steps for the field that will transform eating disorder service provision and facilitate early detection, treatment and recovery for everyone affected by eating disorders, regardless of age, socioeconomic status and personal characteristics.

## Highlights

- Early intervention for eating disorders is in its infancy.
- We summarise existing early intervention approaches for eating disorders from around the globe.
- We propose key policy, service, clinician, and research recommendations to progress early intervention for eating disorders.

**Abbreviations**
CBT‐T: Cognitive Behaviour Therapy‐Ten
DUED: duration of an untreated eating disorder
ED: eating disorders
FREED: First Episode Rapid Early Intervention for Eating DIsorders
UK: United Kingdom
US: United States

## 1 INTRODUCTION

Eating disorders (EDs) are severe psychiatric disorders that affect up to 15% of cisgender women and 5% of cisgender men (Schmidt et al., 2016a; Treasure et al., 2020). Risk is estimated to be 3–4 times higher in transgender and gender non‐binary people (Simone et al., 2022). Existing treatments are moderately effective at best and, on average, only 50% of those with an ED will make a full recovery after treatment (Treasure et al., 2020). This limited recovery rate is coupled with anorexia nervosa having the highest mortality rate of any psychiatric disorder (Schmidt et al., 2016a; Treasure et al., 2020).

Eating disorders have a disease burden comparable to anxiety and depression (Butterfly Foundation for Eating Disorders, 2014), with estimated socioeconomic costs in the billions (USD) each year (Streatfeild et al., 2021). Despite this, ED research is under‐funded compared to other areas of psychiatry (All‐Party Parliamentary Group on Eating Disorders, 2021; Murray et al., 2017; Stone et al., 2021). These minimal research investments have translated to limited opportunities for innovation and system level advancements. Up to 80% of those with an ED do not receive evidence‐based treatment (Kazdin et al., 2017) and early intervention for EDs is not yet well established.

*Early intervention* has been defined as the detection of illness at the *earliest possible point* during the course of a diagnosable disorder, followed by the initiation of stage‐specific, tailored or targeted evidence‐based treatment, which is adapted and sustained for as long as necessary and effective (McGorry et al., 2018). It can be distinguished from *prevention*, which involves intervening *before a diagnosable disorder* is established, often in response to risk markers or initial prodromal symptoms. In many fields, early intervention has led to targeted treatments that give better and more cost‐effective outcomes (McGorry et al., 2008; Shah et al., 2020). In mental health, evidence is greatest for psychosis (Aceituno et al., 2019; Norman et al., 2011) but also exists for mood and anxiety disorders (Anderson et al., 2019; Fineberg et al., 2019). There is initial evidence from the UK that early intervention for EDs can improve outcomes and reduce costs (Austin et al., 2022; Fukutomi et al., 2020). No current consensus exists on what constitutes early intervention for EDs or how this can be tailored to different age groups and care settings. In contrast to psychosis, where the focus may be on facilitating a less severe course of illness, in EDs there needs to be a greater focus on full and sustained recovery.

This paper aims to outline the existing evidence base for early intervention for EDs; provide a global overview of how early intervention for EDs is provided in different regions and settings; and propose key recommendations for policy makers, services, clinicians, and researchers in order to progress early intervention for EDs. The author group brings together leading ED researchers, clinicians and experts by experience from around the globe.

## 2 THE CASE FOR EARLY INTERVENTION FOR EATING DISORDERS

The peak age of onset for an ED is during adolescence and emerging adulthood (i.e., up to age ∼25) (Micali et al., 2013; Solmi et al., 2021; Steinhausen & Jensen, 2015). This is a developmentally sensitive time during which the brain and body are undergoing substantial development and individuals make major life choices (Arnett et al., 2014; Potterton et al., 2020). Developing an ED during this period can lead to long‐lasting consequences for health and life trajectories. In part, this links to neurobiological processes. The brain undergoes considerable development from childhood to adulthood, with most change occurring during adolescence but some continued change into the early‐mid 20s (Mills et al., 2021). Prolonged periods of poor nutrition and stress can disrupt brain development, thereby impacting brain structure and function (King et al., 2018; Walton et al., in press). Evidence from neuroimaging and neurocognitive studies also show that, over time, EDs are associated with changes to brain structure and function that negatively affect chances of recovery (Berner & Marsh, 2014; O'Hara et al., 2015; Steinglass & Walsh, 2016). This has been attributed to habit formation, whereby repeated patterns of behaviour which are initially rewarding become neuro‐behaviourally ingrained, habitual, and less amenable to change (Uniacke et al., 2018; Werthmann et al., 2019).

In keeping with these neurobiological findings, clinical studies show that longer illness duration can be associated with poorer treatment outcomes (Ambwani et al., 2020; Fernandez‐Aranda et al., 2021) and that outcomes may be best during the first 3 years of illness (Ambwani et al., 2020; Austin et al., 2021; Treasure et al., 2015). Results are mixed, however, and a recent meta‐analysis found no significant overall relationship between illness duration and treatment outcome (Redunz et al., 2020). More research is needed, including studies that consider optimal time frames for intervention in different age and diagnostic groups.

There has been a recent call for attention to clinical staging across the spectrum of youth mental health conditions (Shah et al., 2020). There is preliminary evidence to support a staging model of anorexia nervosa, including a longitudinal trajectory from high‐risk markers and prodromal features, to early and later stage illness, with associated neurobiological progression and increasingly muted response to treatment (Maguire et al., 2017; Treasure et al., 2015). There is currently insufficient research to extend a staging model to other EDs.

Most individuals do not access evidence‐based treatment for their ED until many years after they first develop symptoms, if they access treatment at all. *Duration of an untreated ED (DUED)* is the time between first onset of a diagnosable ED and the start of evidence‐based ED treatment. A recent literature review (Austin et al., 2021) found that average DUED ranged from 2.5 years for anorexia nervosa to 6 years for binge eating disorder. These delays in care stem from patient, clinician, service, and system‐level factors. Patient factors include lack of problem awareness and low motivation to change or seek help (ambivalence). Clinician factors include missed detection of EDs or a ‘watch and wait’ attitude. Service and system‐level delays relate to the accessibility and reach of ED treatment. Long waiting times are particularly problematic because they have been linked to worsening symptoms whilst waiting, and poorer outcomes from treatment when it is eventually provided (e.g., Carter et al., 2012; Reichert & Jacobs, 2018). Waiting times for ED treatment have increased further over the COVID‐19 pandemic, with reduced access to health services exacerbated by increased rates of ED symptoms (Devoe et al., 2022).

There are few published studies on efforts to reduce DUED. The Psychenet public health intervention in Germany aimed to address multiple components of DUED via a health literacy campaign, school prevention efforts, specialised anorexia nervosa out‐patient services, a multi‐disciplinary network of health care professionals, and an internet‐based treatment guide. Contrary to expectations, it did not reduce DUED (Gumz et al., 2018), possibly due to small samples, a relatively short implementation period, failure to reach targeted groups, or a lack of intervention effects.

The largest body of research on early intervention in EDs stems from the UK. First Episode Rapid Early Intervention for Eating Disorders (FREED) was developed to provide an early intervention service model and care package for emerging adults (16–25‐year‐olds) with an ED of <3 years duration (Schmidt et al., 2016b). FREED operates within existing ED services, providing a dedicated early intervention pathway alongside usual clinical care. It primarily addresses service‐level components of DUED, although it also aims to promote help‐seeking in young people and prompt referral from primary care. There are waiting time targets of 2 weeks from referral to assessment and 4 weeks from referral to treatment. The FREED care package tailors evidence‐based ED treatment to the developmental needs of emerging adults with a recent‐onset ED. This includes a greater emphasis on engagement and early symptom change; information on the effects of EDs on brain development as a rationale for the importance of early nutritional intervention; exploration of social media use as a potential illness‐maintaining factor; family involvement to support the young person's efforts to change; and attention to ‘emerging adulthood’ and identity formation (Allen et al., 2020). FREED was first evaluated in a single centre pilot study (Brown et al., 2018; McClelland et al., 2018) before being scaled up to four ED services (the FREED‐Up study) (Austin et al., 2022; Flynn et al., 2021). In both studies, FREED was associated with significantly shorter waits for assessment (by ∼3–3.5 weeks) and treatment (by ∼10–12 weeks), compared to treatment‐as‐usual, and shortened DUED by 4–6 months. FREED also led to significant improvements in treatment uptake and clinical outcomes. For FREED patients with anorexia nervosa, 53%–59% reached a BMI >18.5 within 12 months of assessment compared to 17%–18% of treatment‐as‐usual‐controls. The proportion of FREED patients requiring day‐ or in‐patient treatment was also lower (6%–9% vs. 12%–14%), giving cost savings (Allen et al., 2020; Austin et al., 2022; Fukutomi et al., 2020).

The financial argument for early intervention adds to the clinical rationale for this approach. In addition to the direct cost savings demonstrated by FREED, modelling analyses have estimated the long‐term savings that may stem from early intervention for EDs. An Australian report estimated savings of $5 for every $1 spent (Butterfly Foundation, 2014) and a German report estimated savings of €2–4 for every €1 spent on anorexia nervosa and €4 for every €1 spent on bulimia nervosa (Bode et al., 2017). Analyses attended to health care use (including hospital admissions) but also workplace productivity and quality‐adjusted life years.

## 3 EARLY INTERVENTION IN PRACTICE

Most ED treatment guidelines recommend that care is led by a specialist, multidisciplinary team and that outpatient treatment is favoured as a first line option (vs. more intensive treatment) (Hilbert et al., 2017). Beyond this, there is considerable variation in how ED treatment is provided. Referral routes into care, available resources and waiting times, treatment settings, duration of treatment, and funding/insurance processes all vary within and across countries. This section provides examples of how early intervention for EDs is being provided in different geographical regions. These are limited to those countries where early intervention has been studied (primarily WEIRD: Western, Educated, Industrialised, Rich and Democratic). We also provide two lived experience case vignettes, below these geographical examples, which illustrate some of the differences that can occur when early intervention is, and is not, provided.

### 3.1 United Kingdom (UK)

In the UK there is consensus that individuals with EDs should receive treatment, care and support as soon as possible (NHS England with NICE and the National Collaborating Centre for Mental Health, 2019; NICE, 2017; Royal College of Psychiatrists, 2019). For children and young people <18 years, a focus on early intervention is embedded in waiting time targets: 1 week from referral to evidence‐based treatment for urgent cases, and 4 weeks for non‐urgent cases (NHS England, 2015). Services in England have received investment to facilitate these waiting time targets and create a network of specialist, multidisciplinary child and adolescent ED teams. There is an emphasis on effective links between primary care and the specialist ED services; smooth transition procedures between ED services; and personalised evidence‐based treatment planning that attends to the needs of the young person as well as their family/carers (e.g., through providing family support). Self‐referral (or family referral) is possible in some services. Prior to the Covid‐19 pandemic, adherence to waiting time targets approached 90% for both urgent and non‐urgent cases. Latest figures are lower, 59% for urgent referrals and 66% for non‐urgent, likely due to the impact of the pandemic (NHS England, 2022).

Adult ED services in England have not received the same level of investment as children and young people's services, and most are not able to accept self‐referrals. Adults with EDs usually require referral to a specialist service by their primary care doctor (general practitioner; GP) and waits of over 6 months from referral to treatment are common (BEAT, 2018). However, progress is being made via the introduction of FREED. As noted, FREED is a service model and care package for 16 to 25‐year‐olds with an ED of up to 3 years duration. Since the initial pilot and scaling‐up evaluations of FREED (Allen et al., 2020; Austin et al., 2022; Brown et al., 2018; Fukutomi et al., 2020; McClelland et al., 2018), the model has been scaled around England via investment from the National Health Service (NHS) and Academic Health Science Networks. FREED is now being implemented in approximately 80% of adult NHS ED services in England. The impacts of the Covid‐19 pandemic, together with stretched resources for adult ED care in the UK, mean that current adherence to FREED waiting time targets is ∼50% across all services (Richards et al., 2022).

Early intervention for adults over age 25 is lacking in the UK. Early intervention initiatives are also heavily focussed on England (vs. Wales, Scotland and Northern Ireland) and tied to specialist community‐based ED services. University outreach is available in some regions and the UK ED charity, Beat, is trialling a ‘Synergy Support Programme’ to offer individual and family support to under 18's with an emerging ED.

### 3.2 Australia

The UK and Australian health systems resemble each other in terms of a robust public health system closely linked with primary health care workers such as GPs. In Australia, however, there is a greater reliance on private health care alongside the government funded systems. In 2006, private psychological treatment services for people with mental health disorders were included under Australia's Medicare system for the first time, meaning these treatment options became subsidised by the Federal Government. In 2019, expanded Australian Medicare items were made available for EDs. These enable a rebate for up to 40 sessions of evidence‐based therapy and 20 dietetic sessions over a 12‐month period, for patients with anorexia nervosa and patients who meet criteria for a severe ED (Wade et al., 2021). Given the emphasis on severe EDs, the private system is unlikely to have much impact on the provision of early intervention services, thus relegating such approaches to the government sector.

Early intervention has been affirmed as the first of eight critical ED treatment principles in Australia (Heruc et al., 2020). A modified FREED approach has been set up in South Australia through the primary mental health services provided by headspace, showing large effect size decreases in disordered eating following treatment with lower socioeconomic populations (Radunz et al., 2021). The Federal Government has issued a tender for training all workers across primary mental health settings (headspace and its adult counterpart, Head to Health) which may be an important step in developing a critical mass of early intervention services to counter the impact of COVID‐19. There are well‐documented links between COVID‐19 and an increase in disordered eating, EDs, and waiting lists for public ED services around Australia (Springall et al., 2022; Zhou & Wade, 2021).

There is also a focus on offering brief interventions to people on the waitlist for ED services. Not only does this represent a form of early intervention, but it is likely to increase engagement in later treatment (Carter et al., 2012). The earliest evaluation of such an approach (Fursland et al., 2018) offered a Single Session Intervention (SSI) to patients after referral, consisting of an ED assessment plus psychoeducation, delivered face‐to‐face and in writing. Compared to the previous approach of being placed on a waitlist only, this led to an increased uptake of subsequent assessment/treatment and improvements in ED and comorbid symptoms and quality of life. Clinicians around Australia are currently being trained in SSI. A variety of other waitlist interventions are under evaluation, including groups for parents to develop skills in refeeding their child (Eshkevari et al., 2022), online guided self‐help for Family Based Therapy (Wade et al., 2022), online guided self‐help, and an online carer coaching programme at Eating Disorders Victoria.

### 3.3 Canada

Canada's public health care system is provincially and territorially mandated, with vast differences in ED services across the 10 provinces and three territories. Specialised ED services, when available, are often concentrated in intensive settings in major urban cities, leading to limited treatment spaces and long waitlists. Youth in Canada with mild or early‐stage EDs, and those living outside major cities, often lack access to specialised care (Lebow et al., 2021) and any possibility for early detection and intervention. One solution being considered is the spreading of ED services into the community via primary mental health care settings. This is similar to the approach taken with headspace in South Australia. Integrated youth services (IYS) in Canada combine mental health, substance use, primary care, peer support, employment, education and other health, community and social services into an easily‐accessed, youth‐friendly and equity‐focussed service delivery model. They are built on the same principles as early intervention in psychosis services, that is, they emphasise community training for early detection, easy access, early engagement, and illness‐stage appropriate care (McGorry et al., 2008). Leaning on evidence emanating from the FREED model in the UK and headspace clinics in Australia (Radunz et al., 2021), there is a focus in Canada on expanding the IYS provision to EDs. Some early investments in piloting the Canadian adaptation of the FREED model in one province via three IYS sites is currently underway.

### 3.4 United States (US)

In the US, insurance provision is a key determinant of access to health care. There are commercial and government‐funded insurance plans, which vary by state and in the treatment that is covered. This creates a major barrier to early intervention for those without sufficient insurance coverage. There are well‐documented major inequalities in access to health care generally, and ED treatment specifically, within the US (e.g., Kazdin et al., 2017).

Low intensity and community‐based programmes are one way of overcoming some insurance restrictions and improving access to ED treatment across the US. An example is interpersonal psychotherapy, an evidence‐based treatment for binge eating disorder and bulimia nervosa, being provided through college counselling centres. A ‘train the trainer’ model allows continued training of college counsellors within each setting as required (Wilfley et al., 2020). The Body Project prevention programme and associated Body Project Treatment have also improved access to care in educational settings (Becker & Stice, 2017). The original Body Project aimed to prevent onset of an ED amongst women and girls with body dissatisfaction, using cognitive dissonance activities to help dismantle internalisations of body dissatisfaction and reduce thin‐ideal internalisation and dieting. A variant of the Body Project for those with established EDs has also been developed, known as the Body Project Treatment (Stice et al., 2015). This involves eight weekly, 1‐h group therapy sessions focussed on decreasing thin‐ideal internalisation and producing dissonance around body image concerns and ED behaviours. The treatment significantly reduces ED symptoms and facilitates full remission from an ED (Stice et al., 2015, 2019). The Healthy Body Image (Fitzsimmons‐Craft et al., 2019) and StudentBodies/StudentBodies‐EDs (Saekow et al., 2015) programmes are additional online early intervention initiatives that have been evaluated in US schools and colleges. Both draw on cognitive‐behavioural therapy principles. Like the Body Project, StudentBodies focuses on the *prevention* of ED symptoms whereas StudentBodies‐EDs has an early intervention treatment focus. The StudentBodies/StudentBodies‐ED programs have also been used with positive effects in Germany (Saekow et al., 2015). These interventions were developed to be accessible and inexpensive and delivered by a variety of facilitators after standardised training.

Collectively, these initiatives allow good support to be provided to school and college students, but there is a clear need to expand reach to other populations. One study trialled the Body Project with adolescent girls (*n* = 66) in primary care settings and found the programme produced significant reductions in ED symptoms (Linville et al., 2015). This use of primary care fits with efforts in Australia and Canada to integrate early intervention for EDs into primary care hubs. However, primary care provision in the US is also unequal across ethnic and cultural groups (Becker et al., 2003). Universal access to affordable healthcare is needed, combined with active outreach to underserved marginalised groups plus adaptation of care to the needs of these groups.

A recent positive development stems from Project HEAL, which is the leading non‐profit organisation in the US focussed on equitable access to ED treatment. In April 2022, Project HEAL announced the launch of a new Clinical Assessment Programme to provide free, impartial, and culturally competent screening, diagnosis, and onward referral for individuals struggling with disordered eating. This will be provided regardless of the individual's insurance, financial capacity, race, gender, age, treatment history, or co‐occurring diagnoses.

### 3.5 Europe

Nearly all countries in continental Europe have universal health coverage, although the way this is implemented may vary. Online treatment has received attention across Europe, particularly for adolescents and emerging adults. For example, one Dutch trial found a fully automated internet‐based self‐monitoring and feedback intervention was able to significantly reduce ED symptoms over an 8‐week intervention and for up to 6 months thereafter (Aardoom et al., 2016). The sample (*n* = 354) was primarily women aged between 16 and 30 years. Results were strongest for symptoms of bulimia nervosa/binge eating compared to those of anorexia nervosa. As noted, StudentBodies and StudentBodies‐EDs have also been used with positive outcomes in Germany (Saekow et al., 2015). The European Union funded ProYouth initiative is an ongoing online project that aims to promote help‐seeking for EDs and increase healthy eating and body satisfaction amongst 15 to 25‐year‐olds (Bauer et al., 2019). The ProYouth programme includes psychoeducation, feedback on ED symptoms, peer‐to‐peer support, and optional online contact with a psychologist.

There is also an ongoing randomised controlled trial across Germany and the UK to evaluate the effectiveness of online self‐help (EveryBody Plus) for adult women awaiting outpatient psychological therapy for bulimia nervosa or binge eating disorder (Vollert et al., 2018). Qualitative research from the same German‐UK collaboration has found that online self‐help is seen positively as a way of overcoming some of the barriers to seeking and accessing ED treatment (Yim et al., 2021).

### 3.6 Lived experience vignettes

#### 3.6.1 Vignette one

I was 21 and had just moved to a new city when my eating problems took over my life and developed into a full‐blown eating disorder. I struggled for months, knowing that I wasn't coping and that things were getting worse, but not wanting to get better because of the intense fear of gaining weight. My sister eventually persuaded me to go the doctor. Luckily this doctor decided to refer me to a specialist clinic and my recovery journey started from there. Within 2 weeks of my initial referral, I had started a 20 weeks course of CBT with an eating disorder service.

I cannot explain how valuable the early intervention I received has been. Having taken the scary leap to talk to a doctor, not knowing whether I really wanted to recover, being swiftly assessed and diagnosed gave me a validation for my illness. I understood what I had been experiencing and now I knew that clinicians did also. I could explain to others what was ‘wrong’ with me, as the doctors had given me a diagnosis rather than leaving me on a waiting list to receive one. It helped me confirm to myself that my decision to get help had been the right one.

I am now 27 and can say that my eating disorder, whilst it sometimes rears its head and makes an appearance, is now firmly behind me. Without my early intervention treatment and the support from my clinic, I have little idea of the route my life would have taken, but I can safely say that it would not be as fulfiling as the life as I lead now, without that freedom from my eating disorder.

#### 3.6.2 Vignette two

I developed an eating disorder at the age of 8, characterised by food and liquid restriction, over‐exercise, and self‐harm. Disordered eating was a way of coping with early traumas, gastrointestinal problems and emotional distress. I also experienced significant distress in puberty which led to a more severe deterioration in my eating. In later adolescence, it became clear that this body‐related distress and greater eating disorder severity was associated with my non‐binary gender identity, gender dysphoria, and experiences of discrimination, bullying and violence. After a suicide attempt at the age of 14, I was hospitalised and then entered a child and youth mental health service where I was treated for 3 years. I was not diagnosed or treated for an eating disorder until the age of 18 when I became severely ill and was hospitalised with organ failure and a critically low weight. My autism and its relationship to my eating disorder also went unrecognised, which was a significant gap in my treatment. In my late 20s, after multiple repeated therapies, hospitalisations and medications, I began work with a psychologist who approached treatment collaboratively and from an identity‐affirmative and strengths‐based model. For the first time in 22 years of illness, I began experiencing a transformation. I believe that if I had received early diagnosis, access to early treatment, and recognition of my specific needs within treatment, my eating disorder would not have been as severe or prolonged.

## 4 KEY RECOMMENDATIONS

Building on the geographical and case examples above, we propose key recommendations for services and policy makers, clinicians, and researchers. Table 1 provides a summary.

**TABLE 1:** Key recommendations to progress early intervention for eating disorders

Recommendation	Possible facilitators
1. Services and policy makers	
1.1 Equitable access to early, evidence‐based eating disorder care	Provision of eating disorder treatment regardless of disorder stage/severity Provision of care in varied settings including outreach/community‐based services as well as specialist/tertiary services
1.2 Training a diverse workforce that can meet the needs of individuals with eating disorders in the local population	‘Task sharing’ of roles with mental health professionals working alongside experts by experience/peer support workers/low intensity therapists Involvement of carers in service provision Attention to staff characteristics with efforts to promote diversity and inclusivity
1.3 Youth‐friendly care that bridges the 18‐year age divide	Services that work across the adolescent/adult age divide Outreach to relevant community services/sectors, to facilitate easy access into care and smooth transitions out of care Good transition pathways between services
1.4 Flexible treatment delivery—tailoring treatment provision to individual and local needs	Provision of care in varied settings including outreach/community‐based services as well as specialist/tertiary services Use of online, digital, and self‐help interventions
2. Clinicians	
2.1 Early detection of eating disorders	Pro‐active screening and assessment for eating disorder symptoms with prompt onward referral for support when indicated Direct emphasis on the benefits of early intervention including brain changes
2.2 Culturally sensitive care	Clinician training that challenges sociocultural biases relating to eating disorders, weight, health and body image Pro‐active outreach to under‐represented and marginalised groups
2.3 Individualised, developmentally appropriate assessment and treatment	Early provision of relevant psychoeducation, tailored to age and illness stage Positive, recovery focussed, motivational clinician stance that balances attention to ambivalence with an emphasis on the benefits of early intervention and change Routine involvement of family/close others, including the provision of carer support Consideration of social media use, transitions, identity formation and life stage (adolescence/emerging adulthood) Adaptation of evidence‐based treatments to age, illness stage and circumstances with use of online, digital or self‐help formats if applicable
3. Researchers	
3.1 Evaluation of links between DUED and outcomes	Routine assessment of DUED, including further validation of self‐report questionnaires Consideration of the most effective time frame/s for early intervention, by age of onset
3.2 Continued evaluation of early intervention service models and treatments	Assessment of early intervention service models (e.g., FREED) across different settings and populations, including long‐term follow‐up Qualitative and quantitative evaluation of eating disorder treatments within early intervention samples Consideration of developmentally appropriate care including ways to sensitively consider gender identity and sexuality in adolescence and emerging adulthood Multi‐modal neurobiological studies to characterise biomarkers/predictors associated with first episode eating disorders

Abbreviation: DUED, Duration of Untreated Eating Disorder.

### 4.1 Services and policy makers

The above sections highlight considerable variation in how ED treatment is accessed and provided worldwide. A core global principle must be equitable access to early, evidence‐based ED interventions. In some countries, access to treatment is dependent on insurance provision and/or other forms of ‘gate keeping’ procedures around diagnosis, such as a preliminary assessment by a primary health care professional. These processes may delay care and prevent treatment in the early stage of an ED if symptoms are deemed ‘too early’ or ‘too mild’. Changing processes to allow and promote early intervention, with an understanding of the likely long‐term cost benefits, will require policy change. This may include government funding for the full spectrum of ED presentations and interventions being provided in primary as well as secondary/tertiary health care settings, as is starting to occur in some regions.

Early intervention depends on there being enough clinicians available to provide evidence‐based ED treatments. Increasingly, there is a shift to ‘task sharing’ (Kazdin et al., 2017), that is, combining highly trained specialist health professionals (who are often limited in number) with other experts, including ‘experts by experience’, peer support workers, and therapists trained in manualised lower intensity treatments. This mix of staff may allow sufficient overall resources, and bring greater diversity in style, background and experience, with the possibility of a workforce that is more representative of the community it serves. It is also important that services consider support for carers and involve carers as part of ‘expert by experience’ groups.

There is often a division between child/adolescent (<18 years) and adult services despite convincing economic, physical and mental health arguments for youth mental health care that spans this age divide (McGorry et al., 2022). An early intervention ED service needs to provide continuous care to the population most at risk for an ED developing, that is, 12 to 25‐years‐olds. Equally, attention must be given to early intervention across the lifespan, including early intervention for adults who develop an ED beyond age 25, as has been done in psychosis. Services should seek to facilitate swift entry into care through active outreach, self‐referral options, and strong links with other relevant organisations and settings (e.g., schools, universities, youth services, primary care, and other physical/mental health services).

Flexible ways of providing care will support access and may reduce the stigma associated with traditional mental health services. McGorry et al. (2022) argue that a needs‐based approach reduces labelling and over‐diagnosis in young people. As in Australia and Canada, this may include ‘hubs’ located across communities or in university settings, as well as the traditional model of a specialist ED service that covers a large geographical region. Different regions, communities and patient groups are likely to require different approaches to service provision. The COVID‐19 pandemic has accelerated the use of online delivery via video/telehealth and this offers another option for flexible treatment delivery. Early reports suggest that virtual treatment can be as effective as in‐person treatment (Linardon et al., 2022). Indeed, the pandemic may lead to a revolution in digital health care (Barr Taylor et al., 2020) enabling the development of innovative and blended programmes with a wider reach. This will require a pivot in existing approaches, both at the level of the clinician and systemic regulatory frameworks (Barr Taylor et al., 2020; Waller et al., 2020).

### 4.2 Clinicians

Assessment of first episode EDs requires a holistic, individual approach that considers developmental and illness stage (Schmidt et al., 2016b). This will facilitate treatment planning that is developmentally informed, individualised, and evidence‐based. As EDs typically develop during adolescence and emerging adulthood, clinicians need to understand this developmental period. Within the FREED model, clinicians are advised to pay particular attention to social media use as a potential maintaining factor, stresses around identity/emerging adulthood and transitions, and support from family or close others (Allen et al., 2020).

Eating disorders, particularly anorexia nervosa, may be ego‐syntonic and those with recent onset may be especially ambivalent regarding recovery, given that some of the negatives of the ED may not have become apparent at initial stages. The clinician must skilfully balance acknowledgement of ambivalence with the importance of rapid recovery (Mills et al., 2021). Co‐morbidity between EDs and other mental health difficulties may add to treatment challenges, and using available resources (e.g., Tchanturia, 2021, for autism) will help with navigating these.

Early intervention through or with families/carers deserves consideration, particularly given the effectiveness of family‐based interventions for adolescents with EDs (Lock & Le Grange, 2019). Families are often best placed to spot emerging ED symptoms and parents may be more motivated to seek early intervention than young people themselves. Levels of carer distress and burnout are well documented (Anastasiadou et al., 2914; Zabala et al., 2009) and Schmidt and Treasure (2006) hypothesised that carers may inadvertently engage in enabling or accommodating behaviours that risk strengthening the ED. There is good evidence that parent skills training groups can increase carer self‐efficacy whilst reducing distress, anxiety and burden (Macdonald et al., 2011; Nicholls & Yi, 2012; Rosello et al., 2021).

Within many regions, efforts to deliver early intervention are hampered by long waiting times, relatively high levels of drop out (although FREED has demonstrated lower drop‐out rates than treatment‐as‐usual) and an often transient population (e.g., moving geographically to attend university or for work/travel). Creative solutions are needed to tackle such challenges. Online programs and virtual working are possible approaches and examples from the US and Europe offer evidence‐based solutions in this area. Another approach is using brief interventions that can be delivered by facilitators of variable expertise under expert supervision. The 8‐session Body Project Treatment is one such example (Stice et al., 2019). Another is Cognitive Behaviour Therapy–Ten (CBT‐T), a 10‐session adapted version of CBT‐ED that is suitable for individuals with non‐underweight EDs (Waller et al., 2019). CBT‐T has demonstrated effectiveness across varied healthcare settings (e.g., Rose et al., 2021; Tatham et al., 2020). Guided self‐help also has a strong evidence base for binge/purge type EDs and can be delivered flexibly, in different formats, and with varying ‘doses’ of support including online only delivery, telephone or video supporting calls, or face‐to‐face support (Traviss‐Turner et al., 2017; Yim & Schmidt, 2019). There is good evidence that guided self‐help is an acceptable, effective and cost‐effective option in the treatment of adolescents and emerging adults with binge/purge EDs (Pretorius et al., 2009; Schmidt et al., 2007).

Attention to diversity and inclusion is key so that all individuals with EDs can access support that is appropriate and sensitive to their needs and characteristics. There is still much work to be done to ensure that early intervention programs are applicable to racial, ethnic, and gender minorities. This is a global issue but US studies shed light on how detection of ED symptoms may vary across groups. One US study of over 9000 participants assessed how ethnicity impacts access to care for ED symptoms. Clinician bias was thought to result in Black, Latina/o and Native American participants being significantly less likely to seek care or to receive recommendations for further evaluation than White participants (Becker et al., 2003). Clinicians with a higher emphasis on health may also be more likely to miss an ED (Worsfold & Sheffield, 2020). Such research highlights the need for comprehensive training that uses an intersectional lens, in order to challenge long held and mass perpetuated ideals.

More broadly, all of the clinician recommendations offered here rely on clinical training that gives sufficient attention to the detection, diagnosis and management of eating disorders. This needs to extend to primary care practitioners and general physicians as well as mental health specialists. It has been demonstrated that these groups receive limited specialist training in EDs (Ayton & Ibrahim, 2018). Early intervention depends on early detection, and missed opportunities due to inadequate training, or sociocultural or clinician biases, will hamper progress for the field.

### 4.3 Research

Research into early intervention in ED is in its infancy. As a starting point, and given inconsistent links between DUED and treatment outcome, there is a need for additional research on how to operationalise and define ‘early intervention’. The gold standard assessment of DUED would involve an interview, with autobiographical events used as anchor points to assess the onset and change of ED symptoms and help‐seeking behaviours. However, self‐report questionnaire measures of DUED would be easier to use and validating these may facilitate more widespread assessment of DUED in clinical and research settings. This, in turn, would facilitate more research on how DUED relates to outcomes and the most effective time frame/s for early intervention, which may vary by age of onset.

There is also a need for further research on early intervention service models and care approaches, in terms of their effectiveness, scalability, and scope for implementation across patient groups, services and regions. There are few studies, and no randomised controlled trials to date, examining how evidence‐based ED treatments generalise to an early intervention sample. Quantitative and qualitative evaluation of treatment outcomes in an early intervention context is important, with attention to long‐term effects as well as how carers/family members perceive treatment.

As EDs frequently develop during adolescence, research on developmentally appropriate care is essential. This includes sensitive assessment and treatment processes for young people going through puberty and developing their sense of self and identity, with consideration of gender identity and sexuality, culturally appropriate body image assessments, and experiences of discrimination around size and shape (e.g., Hartman‐Munick et al., 2021; Nowaskie et al., 2021; Parker & Harriger, 2020; Raffoul & Williams, 2021). In 2011, the American Institute of Medicine emphasised research on the needs of sexual minority populations with EDs as being a public health priority. Transgender and gender‐expansive populations are at increased risk for EDs (Simone et al., 2022) and face multiple barriers to ED screening and treatment (i.e., lack of gender‐affirmative care, decreased health care utilisation) that may limit access to effective early intervention (Hartman‐Munick et al., 2021). Early intervention in these populations is complex, as there is a dearth of studies examining the use of a validated ED questionnaires for transgender and gender‐expansive clients, and treatment providers may not receive gender‐affirmative training.

Alongside these clinically focussed studies, multi‐modal neurobiological studies (including genetic, neuroimaging and neurocognitive assessments) characterising first episode cohorts and following them into recovery are needed. These may help to identify biomarkers/predictors for early or delayed recovery and identify those who may be at risk of illness progression and a less favourable course.

There are various encouraging research streams underway that will start to address these needs. Initial investment in Canada has allowed for a focus on early intervention via primary care settings and additional research is planned. In the UK, the 4‐year EDIFY project (Eating disorders: delineating illness and recovery trajectories to inform personalised prevention and early intervention in young people) launched in late 2021. This project brings together researchers, clinicians and young people with the goal of co‐creating an interdisciplinary, evidence‐based model of how EDs develop and are maintained. The FREED team are also piloting a FREED‐on‐Mobile (FREED‐M) app to encourage help‐seeking amongst young people with EDs. The app delivers youth‐friendly psychoeducation via animations and downloadable resources, with the goal of encouraging young people to seek help for their ED. In Australia, calls have been made for a youth‐focussed research agenda for EDs (Allison et al., 2021) including more work across the 18‐year‐old threshold that divides child/adolescent and adult services. In the US, the Strategic Training Initiative for the Prevention of EDs (STRIPED) has called for patient‐centred and community‐based research on barriers to early identification of EDs, treatment seeking, and access to care. As noted, the European ProYouth initiative is an ongoing online project focussed on improving help‐seeking and reducing ED symptoms.

## 5 CONCLUSION

Attending to these recommendations would transform ED service provision and allow early intervention to be a standard part of best practice care. Progress in other areas of psychiatry shows that this is possible. We invite those working in the ED field to mobilise change, so that we can move away from a landscape where up to 80% of those with an ED do not receive treatment (Kazdin et al., 2017) and up to 50% of those who receive treatment remain symptomatic at the end (Treasure et al., 2020).

## Data Availability

Data sharing is not applicable to this article as no new data were created or analysed in this study.
